# Notch signaling promotes the corneal epithelium wound healing

**Published:** 2012-02-09

**Authors:** Huayi Lu, Qingxian Lu, Yajuan Zheng, Qiutang Li

**Affiliations:** 1 Department of Ophthalmology and Visual Sciences, James Graham Brown Cancer Center, University of Louisville School of Medicine, Louisville, KY; 2 Second Hospital of Jilin University, Changchun, Jilin Province, P.R. China

## Abstract

**Purpose:**

The Notch signaling pathway plays crucial roles in regulation of cell proliferation, differentiation and cell fate decision in multiple tissues and cell types. This study was designed to test the effects of enhanced Notch activity on corneal epithelium homeostasis and wound healing using the transgenic mice that overexpressed an activated Notch1 (*NICD*) in cornea epithelium.

**Methods:**

The studies were performed on *R26^fN1-ICD^* transgenic mice that carry a *NICD* cDNA (cDNA) whose expression is prevented by a “Lox-STOP-Lox” cassette. When this transgenic mouse is bred to a mouse strain carrying a Cre recombinase expression cassette driven by a tissue-specific keratin 14 (*K14*) promoter, the floxed “STOP” cassette is excised and *NICD* is expressed in the cornea epithelium. The expression level of *NICD* and its downstream target genes, hairy and enhancer of split 1 (*Hes1*) and hairy/enhancer-of-split related with YRPW motif 1 (*Hey1*), in the transgenic corneal epithelium was examined by quantitative PCR (qPCR). The phenotypes and morphology of the transgenic corneal epithelium were compared with that of wild type (WT) controls. The proliferation rate of the epithelial cells was assessed by 5-bromo-2'-deoxyuridine (BrdU) incorporation and the differentiation statues were examined by K14, tumor protein p63 (p63), K12, and zona occludens 1 (ZO-1) immunoreactivity at either normal developmental condition or after corneal epithelial debridement. The corneal epithelial response to wound healing was studied by fluorescent staining and Richardson’s staining macroscopically and by H&E staining at microscope level at 0, 6, 12, 18, and 24 h post injury.

**Results:**

Although overexpression of *NICD* in cornea epithelium led to upregulation of its downstream targets, i.e., *Hes1* and *Hey1*, this did not alter corneal epithelial cell proliferation and differentiation. However, wound healing induced Notch activity and overexpression of *NICD* promoted corneal epithelial wound healing, which was in agreement with more rapid early proliferation response in *NICD* transgenic mice than in the wild type control mice.

**Conclusions:**

These findings further demonstrate the functional role of Notch signaling in corneal epithelium wound healing response.

## Introduction

Corneal epithelium covers the surface of the anterior eye to protect it from various environmental injuries including physical and chemical insults, and microbial infection. Coping with this specific need, corneal epithelium adapts continuous self renewal ability and fast wound healing response. The well coordinated cell proliferation, migration, differentiation, and cell death are required to maintain both epithelium renewal and wound healing. Five hours after central epithelial wounding, epithelial cells at the wound edge begin to slide horizontally to cover the denuded surface [1]. The cells near the wound edge are mitotic inactive and they migrate from the peripheral area where the cells actively proliferate to continuously provide the demanded epithelial cells until normal epithelium is restored at the wound area. The cells that have migrated to the wound area differentiate properly to form tight junction and reestablish the barrier function. A 1.5 mm epithelial debridement can be healed in 24 h. A prompt recovery from the corneal wound is critical to maintain the cornea barrier that is essential for appropriate vision. Several signaling pathways and growth factors are involved in regulation of corneal epithelial homeostasis and wound healing [2]. However, the precise molecular mechanisms are still not fully understood.

Notch signaling is a key pathway in regulation of cell proliferation, differentiation, and death in multiple tissues and cell types. The Notch family consists of four transmembrane receptor members, specifically Notch1, 2, 3, and 4; there are five ligands for Notch family: Jagged1, Jagged2, Delta1, Delta3, and Delta4 [3]. When engaged with the ligand, Notch releases Notch intracellular domain (NICD). The released NICDs bind to recombination signal binding protein for immunoglobulin kappa J region (Rbpj) in the nuclei and directly regulate expression of multiple downstream targets in a tissue and cell specific manner [3]. Notch signaling is important to maintain the corneal homeostasis. Both Notch1 and Notch2 were detected in the human corneal suprabasal epithelial cell layers, whereas the ligands Delta1 and Jagged1 were observed throughout the corneal epithelium [4]. Active NICD was also detected in the basal and early suprabasal layers in the cornea epithelium, and the increase in Notch activity enhanced corneal epithelial cell proliferation in vitro [5]. Notch1 is required to maintain the corneal epithelial cell fate during wound healing [6]. As a major transcription target gene of Notch signaling, hairy and enhancer of split 1 (*Hes1*) is also critical for corneal development and *Hes1* deficient mice show abnormal cell junction and cell differentiation, and decreased cell proliferation [7].

Notch signaling plays an important role in the regulation of corneal epithelium homeostasis and wound healing response. However, the regulation and function of notch signaling in corneal epithelium in vivo are still not fully characterized. In the current report, the transgenic mice that express an activated NICD in cornea epithelium were used to examine its effects on corneal epithelium homeostasis and wound healing.

## Methods

### Animal model

To create cornea epithelium-specific *NICD* transgenic mice, we crossed *K14-cre^+/+^* transgenic homozygous mice (stock number 004782; derived from B6xCBA F1; Jackson Laboratory, Bar Harbor, ME) with *R26^fN1-ICD^* transgenic mice (Stock number 008159, Jackson Lab) to generate two types of mice, *K14-cre^+/−^/NICD^+^,* and *K14-cre^+/−^* mice (ratio of 1:1) [8,9]. All studies are conformed to ARVO Statement for the Use of Animals in Ophthalmic and Vision Research and the institutional IACUC protocol.

### Cornea epithelium debridement, fluorescein staining, and Richardson’s staining

Adult *K14-cre^+/−^/NICD^+^, K14-cre^+/−^* and wild type (WT) mice at the ages of 6–10 weeks were selected and anesthetized with an intraperitoneal injection of ketamine (50 mg/kg) and xylazine (5 mg/kg). After a few drops of 0.5% topical proparacaine were applied, the central epithelium, excluding the limbal area, was demarcated with a 1.5-mm-diameter biopsy punch and removed by gently scraping with a blade [10]. This type of wound only removes the epithelium and leaves the basement membrane intact. The epithelial wound was monitored right after injury with the fluorescein staining. Corneal surface integrity was examined and photographed with a slit lamp biomicroscope equipped with a cobalt blue light, after a 2-min surface staining with 2 μl of 0.5% fluorescein sodium salt and intense washout of the extra fluorescein with 1 ml PBS. The wound-healing process after 6, 12, 18, and 24 h was further monitored and recorded using the fluorescein staining method described earlier, after which the animals were euthanized, and the eyes were collected for either immunohistochemistry or isolation of RNA and protein from the corneas. The wound areas were measured in Portable Document Format (PDF) files using the measure tool and expressed in square millimeters (mm^2^). To avoid bias, genotyping of WT and transgenic mice were masked during the wounding experiments, image taking, and wound-area measurements.

In addition, we monitored the wounding area by Richardson’s staining to reveal exposed basement membrane. At 0, 18, and 24 h after epithelium debridement, eyes with wounded cornea in *K14-cre^+/−^/NICD^+^* and *K14-cre^+/−^* mice were fixed in 4% paraformaldehyde (PFA) for 2 h. Four eyes were used for each time point. Whole eyes were stained with Richardson's solution (equal mixture of 1% azure II and 1% methylene blue/1% borax dissolved in 100 ml autoclaved water) to reveal exposed cornea basement membrane. Then corneas were removed and photographed with a digital camera connected to a dissecting microscope. The results are presented as average±SD and a value of p<0.05 was considered to be statistically significant.

### Tissue collection

Corneas for RNA or protein preparation were dissected from WT, *K14-cre^+/−^*, and the *K14-cre^+/−^/NICD^+^* mice under a dissecting microscope. For cell proliferation assay and immunohistochemistry, whole eyes were dissected 2 h after 5-bromo-2'-deoxyuridine (BrdU; 150 mg/kg bodyweight) injection and fixed in 4% PFA at 4 °C for 24 h and followed by routine paraffin-embedding and sectioning procedure. The sections were cut serially at 5 μm.

### Immunohistochemical analysis

Sagittal eye sections (5 μm) were treated with antigen retrieval procedure by boiling in 10 mM Tris-ethylendiaminetetraacetic acid (EDTA) buffer (for 1 liter: 1.2 g Tris and 0.37 g EDTA, the solution was about pH 9) for 30 min, and then slowly cooled down in the same heating buffer at room temperature. After blocking with the blocking buffer (1× PBS, 0.2% Triton X-100, 5% normal serum), the slides were incubated with primary antibody in the blocking buffer for 1 h at room temperature; followed by three washes with PBS, 10 min each; and then by a 30 min incubation with appropriate secondary antibodies. The primary antibodies are rabbit anti-K14 (1:500, Cat# PRB-145P; Covance Research Products, Denver, PA), mouse anti-p63 (1:200, Cat# sc-8431; Santa Cruz Biotechnology, Santa Cruz, CA), goat anti-K12 (1:200, Cat# sc-17101; Santa Cruz Biotechnology), rat anti-BrdU antibody (1:800, Cat# MAS 250c; Harlan-Sera Lab, Belton Loughborough, Leicestershire, England), and rabbit anti-zonula occludens-1 (ZO-1; 1:600, Cat # 61–7300; Zymed Laboratories Inc., San Francisco, CA). The secondary antibodies were conjugated with either Cy3 or FITC and obtained from Jackson ImmunoResearch Laboratories, Inc. (West Grove, PA). Nuclei were stained by diamidinophenylindole (DAPI).

### BrdU- positive cell counts and statistical analysis

On each corneal section, the BrdU-positive cells in corneal and limbal epithelia were enumerated on a digital micrograph taken at 100× magnification. The numbers of BrdU positive cells were divided by the length of corneal or limbal epithelia, respectively, to produce a number of BrdU positive cells per 1 mm. The results were expressed as the average counts from three sections per eye with 4 to10 eyes in each experimental group. All data are shown as the average±standard deviation (SD). The differences were considered statistically significant if the p values were less than 0.05.

### RNA isolation and qPCR

Total RNA was prepared from four eyes in each genotype using TRIzol reagent (Invitrogen, San Diego, CA). The A260/A280 ratio of all RNA samples was >2.0 as measured by NanoDrop 1000 Spectrophotometer Nanodrop (Thermo Fisher, Wilmington, DE). cDNAs were reverse-transcribed using random primers and SuperScript VILO cDNA synthesis kit (Invitrogen). qPCRs were performed in a SYBR green-based PCR reaction mixture on a MX3005p system (Agilent Technologies, Inc., Santa Clara, CA), with a 10-min initial hot-start activation of Taq polymerase at 95 °C and followed by 40 cycles of amplification (95 °C for 25 s, 56 °C for 30 s, and 72 °C for 30 s). The comparative Ct (threshold cycle) method normalized to β-actin (*Actb*) was used to analyze relative changes in gene expression. The qPCR primer sequences for *NICD*, *Hes1*, and *Hey1* were listed in Table 1 and described previously [11].

**Table 1:** List of primers used for qPCR.

**Gene**	**Primer sequence**
Notch1	F-5’- TCAATGTTCGAGGACCAGATG -3’
	R-5’- TCACTGTTGCCTGTCTCAAG -3’
*Hes1*	F-5’- CTACCCCAGCCAGTGTCAAC -3’
	R-5’- ATGCCGGGAGCTATCTTTCT -3’
*Hey1*	F-5’- TACCCAGTGCCTTTGAGAAG -3’
	R-5’- AACCCCAAACTCCGATAGTC -3’

## Results

### *NICD* transgene expression in corneal epithelium does not affect the corneal homeostasis

**Figure 1:**
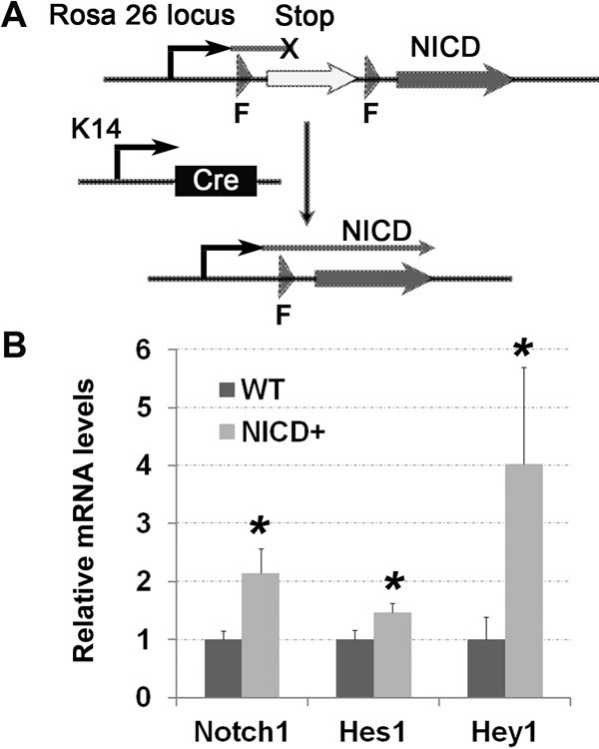
*NICD* transgene expression. **A**: Schematic view of the experimental strategy. *R26^fN1-ICD^* mice were crossed with *K14-cre^+/+^* mice to generate *NICD* overexpression corneal epithelium in *K14-cre^+/−^*/*NICD^+^* mice. F, floxed. **B**: mRNA levels of *NICD* and its direct downstream transcription targets, *Hes1* and *Hey1*, were assessed by a quantitative reverse transcription-PCR analysis of cornea extract RNA from 8-week-old WT and *K14-cre^+/−^*/*NICD^+^* mice. Each bar represents average±standard deviation (SD) of three independent experiments. Two-tail *t*-test: *p<0.05. n=3.

**Figure 2:**
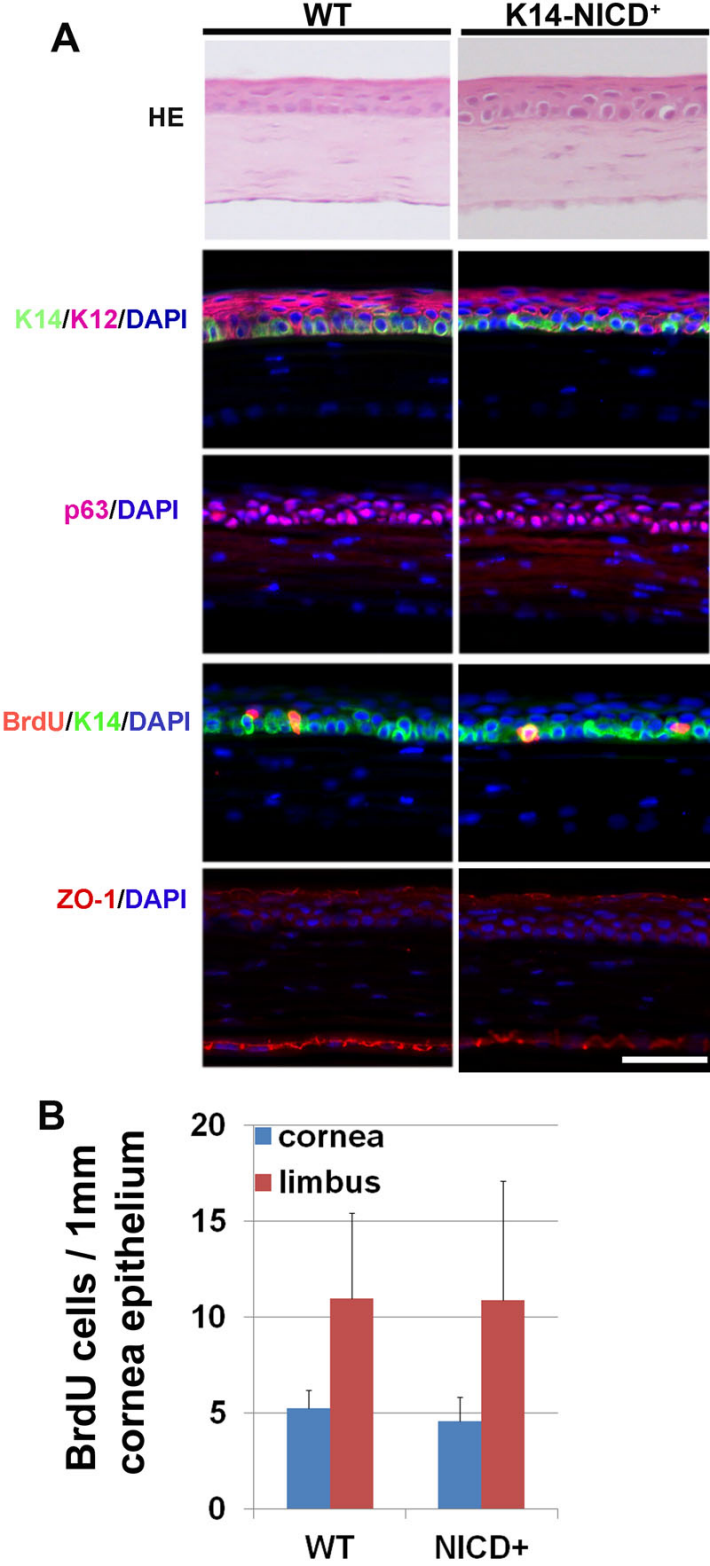
No development defects in *NICD* transgenic cornea. **A**: Immunohistochemistry examination of *NICD* transgenic eye in comparison with wild type control animal. The representative images of HE staining and immunofluorescent staining with antibodies recognizing K14, p63, BrdU, K12, and ZO-1 in the corneas of 8-weeks-old WT and *K14-cre^+/−^*/*NICD^+^* mice. The scale bar represents 50 μm of length. **B**: Quantitative examination showed no significant difference in the cell proliferation between *NICD* transgenic cornea and WT cornea. The average numbers of the BrdU positive cells per 1 mm of fixed length parallel to the cornea surface or limbus±SD were calculated from 5 eyes for each type of mice. n=5.

The Notch signaling pathway is critically involved in cell fate decisions during the development of many tissues and organs. To analyze the effect of *NICD* overexpression on corneal epithelium homeostasis and wound healing, we generated the transgenic mice with cornea epithelium-specific expression of *NICD* cDNA by breeding the previously described *K14-cre^+/+^* and *R26^fN1-ICD^ mice* for the generation of *K14-cre^+/−^*/*NICD^+^* mice (Figure 1A) [8,9]. RT-qPCR consistently showed a higher expression of Notch1 in adult *K14-cre^+/−^*/*NICD^+^* corneas than in WT corneas (Figure 1B). Among the immediate Notch target genes, *Hes1* and *Hey1* were upregulated in *NICD* transgenic corneas (Figure 1B). The newborn *K14-cre^+/−^*/*NICD^+^* pups showed severe skin blistering in the limb area, however, the pups appeared healthy after the first week. The skin blistering in *NICD1* transgenic mice was caused by the loss of integrin expression and consequently, the loss of hemidesmosomes due to *NICD* induction, as described previously [12]. In addition, the *NICD1* transgenic mice showed various proliferation and differentiation defects in the epidermis [12]. Surprisingly, a normal corneal morphogenesis in the transgenic mice at 2 and 4 months old was observed by the hematoxylin and eosin (H&E) staining of paraffin-embedded sections (Figure 2A); further examination of the progenitor markers p63, cytokeratin 14 (K14), K12, and tight junction maker ZO-1 by immunohistochemistry showed no abnormality in corneal epithelium differentiation (Figure 2A). In both WT and *K14-cre^+/−^*/*NICD^+^* transgenic mice, the basal layer maker K14 and p63 were similarly distributed and mostly confined to the corneal basal epithelial layer. K12 was strongly shown in the corneal suprabasal layer cells that normally form tight junctions at the cornea surface as judged by ZO-1 immunostaining (Figure 2A).

To further examine cell proliferation in the cornea epithelium, we injected animals with BrdU, a thymidine analog that incorporates into DNA during the S-phase of the cell cycle. The similar numbers of BrdU labeling cells were observed between *K14-cre^+/−^*/*NICD^+^* transgenic and WT corneas, suggesting that ectopic expression of NICD did not change cell cycling in cornea epithelium (Figure 2B).

### *NICD* expression promotes corneal epithelial wound healing

**Figure 3:**
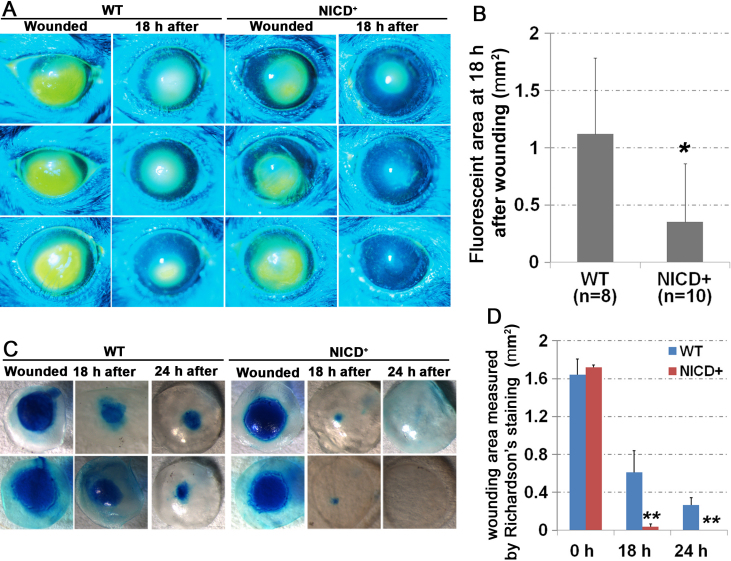
Fast recovery from cornea wounding in *K14-cre^+/−^*/*NICD^+^* transgenic mice. **A**: The fluorescein stained eye images were taken under cobalt-filtered ultraviolet light right after, or 18 h after, mechanical scraping of the center corneal epithelium. **B**: Quantitative measurement of wounding area stained by fluorescein is presented as average wound area per eye. “N” in the figure represents the number of eyes examined. Two-tail *t*-test: *p<0.05. **C**: Wounded corneas were stained with Richardson’s solution to show the remaining wound area (blue at 0, 18, and 24 h after injury). **D**: The wound size (Richardson stained region) was measured using photographs with computer image software. Four eyes for each group and the results are presented as average±SD.. Two-tail *t*-test: **p<0.005 and *p<0.05. n=4.

The process of cell proliferation, differentiation, and migration during corneal wound healing response is highly coordinated and precisely controlled, and Notch plays a role in those events. We therefore examined the effect of ectopic expression of NICD on the corneal epithelial debridement wound healing. Mice at 2 months of age were wounded by a corneal debridement procedure. A 1.5-mm diameter circle at the central epithelium, excluding the limbal region, was removed. The corneal surface wound was then observed and monitored by fluorescein dye. Based on the measurement of fluorescein stained area at 18 h and 24 h after injury, we found that overexpression of NICD in corneal epithelium promoted wound healing process (Figure 3A,B). Eighteen hours post injury, the wound area was significantly reduced in the *K14-Cre^+/−^*/*NICD^+^* transgenic mice compared to the age matched WT mice, as assessed by fluorescein staining (0.355 mm^2^ [n=10] versus 1.121 mm^2^ [n=8]; p<0.05; Figure 3A,B).

We further confirmed the results with a better staining technique, Richardson's staining, to quantify the healing rate at 18 h and 24 h after injury. After the removal of central cornea epithelium demarcated with a 1.5 mm biopsy punch, the initial wound areas in wild type control mice (1.71 mm^2^) and *NICD* transgenic mice (1.70 mm^2^) were similarly produced as determined by the Richardson’s stained cornea (Figure 3C,D). However, the remaining wound area at 18 h after wounding in *NICD* transgenic corneas was significantly less than in controls (0.036 mm^2^ [n=4] versus 0.612 mm^2^ [n=4]; p<0.005; Figure 3C,D). At 24 h after debridement, the wounds were completely healed in *NICD* transgenic mice, while the wound areas were still present at the wounding site in WT cornea (0 mm^2^ [n=4] versus 0.263 mm^2^ [n=4]; p<0.005; Figure 3C,D). The results indicate that *NICD* overexpression in vivo enhances epithelial wound closure.

### *NICD* overexpression alters the kinetics of corneal epithelial proliferation in response to wounding

**Figure 4:**
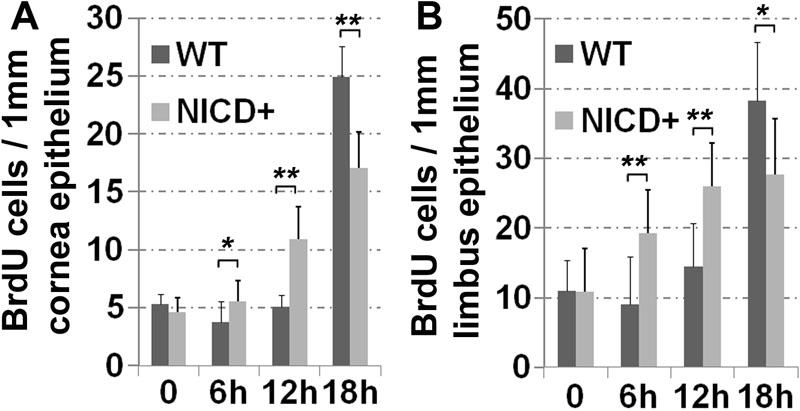
Increased cell proliferation at earlier responses to wounding in *K14-cre^+/−^*/*NICD^+^* cornea. **A**: Quantification of BrdU positive cells in the corneal epithelium of the WT and *K14-cre^+/−^*/*NICD^+^* mice at different time points after wounding. The average number of the BrdU positive cells per 1 mm length parallel to the cornea surface was calculated from the counts on three random sections of each four wounded eyes. **B**: Quantification of BrdU positive cells in the limbal epithelium of the WT and *K14-cre^+/−^*/*NICD^+^* mice at different time points after wounding. Each bar represents the average number of the BrdU^+^ cells per 1 mm limbal epithelium±SD. The number was calculated from the counts on three random sections from each four wounded eyes for each experimental group. Two-tail *t*-test: **p<0.005 and *p<0.05. n=12.

To further dissect the cellular mechanisms underlying the promotion of corneal epithelial debridement wound healing by active nuclear NICD, we first examined the dynamics of cell proliferation in response to epithelial debridement wounding, using BrdU labeling and immunostaining quantification. The incorporated BrdU was primarily localized in the basal corneal cell layer. After wounding, the BrdU positive cells were gradually increased in a time window of 12 to 18 h post surgery in the WT corneal epithelium (Figure 4A). However, the kinetics of cell proliferation in the transgenic corneal epithelium was dramatically shifted, with a rapid increase in the number of BrdU labeled cells in the period of 6 to 12 h after wounding, as compared to the WT control (Figure 4A). The average number of BrdU-positive cells in a fixed length of transgenic cornea epithelium on 5 μm sagittal corneal sections was doubled to that of the WT controls during the same period following injury (Figure 4). However, by 18 h after wounding, fewer BrdU-labeled cells were observed in the *NICD* transgenic mice than in the WT animals. Such proliferation kinetic patterns were also observed in the limbal areas. Those data suggest that active Notch promotes rapid cell proliferative response.

### Notch1 expression and its activity in WT mice are upregulated after wounding, correlating with proliferation induction

**Figure 5:**
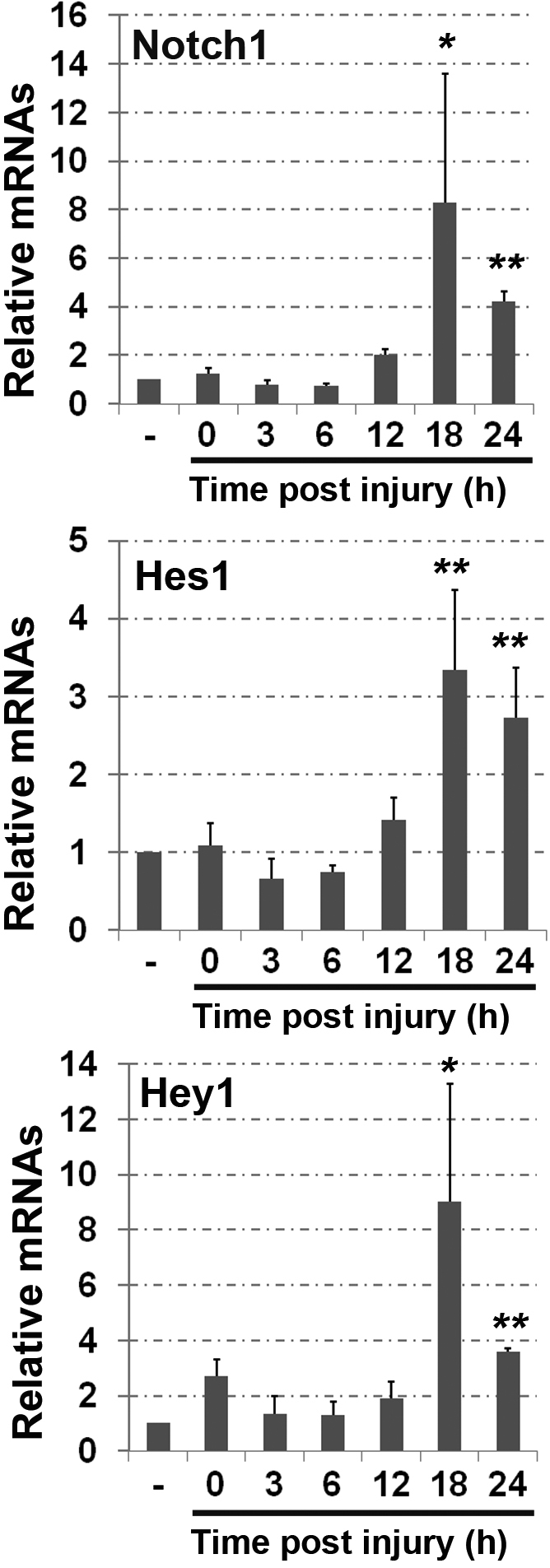
Upregulation of *Notch1*, *Hes1*, and *Hey1* during wound healing response in WT mice. Rea-time qPCR analysis of *Notch1*, *Hes1*, and *Hey1* mRNA expression performed in WT cornea tissues collected from no-injured corneas and corneas at 0, 3, 6, 12, 18, and 24 h following injury. Each bar represents average±SD of 4 independent experiments. Two-tail *t*-test: **p<0.005 and *p<0.05. n=4.

The results shown above suggest that increased Notch activity can promote cell proliferation in the wound healing response. To further correlate corneal epithelial cell proliferation initiated by wounding in normal mice with expression and activity of Notch, we examined mRNA levels for *Notch1*, *Hes1*, and *Hey1* during the process of wound healing. Corneas from WT mice at 0, 3, 6, 12, 18, or 24 h after debridement were used for RNA isolation and qPCR quantification. No significant change was observed in the mRNA levels of *Notch1*, *Hes1*, and *Hey1* at the time points of 0, 3, 6 or 12 h after debridement. However, by 18 to 24 h after injury, *Notch1*, *Hes1*, and *Hey1* mRNAs were significantly increased as shown by real time qPCR assays (Figure 5). The induced Notch activity (Figure 5) was correlated to the BrdU incorporation pattern with the peak cell proliferation response starting at 18 h after injury (Figure 4). These observations support the hypothesis that an increased Notch activity during wound healing process contributes to corneal epithelial proliferation response.

## Discussion

Corneal integrity is critical for normal vision. The cornea epithelium, located on the anterior eye, is constantly exposed to chemical and physical insults. A rapid and efficient healing from those environmental damages is necessary to maintain a normal vision; this healing process is precisely coordinated by a serial of highly controlled events, such as cell migration, proliferation, and differentiation, which are mediated through several growth factors and molecular signaling pathways. In the current investigation, we showed that the Notch signaling pathway was important for modulation of the wounding response. Overexpression of active NICD allowed the corneal epithelial cells to respond to wounding more rapidly and promoted an earlier and quicker cell proliferation response by the transgenic cornea, as compared to the WT cornea epithelium.

The Notch signaling pathway is critical for cell fate decisions during development and wound healing [6]. Activation of Notch signaling in keratinocytes is sufficient to cause cell cycle withdrawal and trigger terminal differentiation [13]. Interestingly, overexpression of active NICD in corneal epithelium in vivo did not cause significant abnormality in corneal epithelial proliferation and differentiation under normal physiologic condition. However, the enhanced Notch activity efficiently promoted the corneal wound healing by stimulation of a rapid early cell proliferation. This finding was in agreement with previous studies showing that inhibition of Notch activity in mice significantly delayed the healing of dermal wounds, and activation of Notch activity in vivo boosted wound repairing [14]. Our data suggest that Notch signaling plays a beneficial role in corneal epithelial debridement wound healing by increasing transient cell proliferation.

The mechanism by which Notch contributes to cell proliferation remains elusive and Notch displays dichotomous functional activities, promoting both proliferation and growth arrest. It has been reported that activation of Notch signaling in keratinocytes is sufficient to cause cell cycle withdrawal through the induction of p21 and promote the entry of terminal differentiation in the intermediate epidermal layers [13]. However, the effects of Notch on cell proliferation appear to be dependent on tissue and dosage. In the corneal wound model, *Hes1* expression has been associated with the cell proliferation activity in vivo, while overexpression of *Hes1* in a 3D corneal culture model was shown to suppress cell proliferation [7]. Moreover, dose-dependent induction of distinct phenotypic responses to Notch pathway activation was observed in mammary epithelial cells [15]. The expression of a constitutively active NICD was found to induce two distinct types of 3D structures of MCF-10A mammary epithelial cells: large, hyperproliferative structures and small, growth-arrested structures with reduced cell-to-matrix adhesion [15]. High Notch activity caused down-regulation of multiple matrix-adhesion genes and inhibition of proliferation, whereas low Notch activity maintained matrix adhesion and provoked a strong hyperproliferative response. In cornea wound healing model, we observed the induced Notch1 activity associated with cell proliferation activity in the cornea epithelium, indicating that Notch1 plays an important role in inducing corneal epithelial cell proliferation. However, overexpression of *NICD* does not affect the normal corneal epithelial proliferation, suggesting that other wounding response factors may contribute to the sensitivity of cells responding to Notch activity.
